# Perioperative risk factors and risk modeling for frailty one month after spine surgery in elderly patients

**DOI:** 10.1186/s12891-026-09934-3

**Published:** 2026-06-01

**Authors:** Yao Wang, Pingxiu Zhang, Yurong Zheng, Xiaobo Wang, Congrui Liao, Meiying Li, Wanyi Jiang, Huiqing Li, Xiaohuan Yi, Zhongmin Zhang, Lan Feng

**Affiliations:** Department of Spine Surgery, Nanfang Hospital, Southern Medical University, Guangzhou, China

**Keywords:** Spine Surgery, Frailty, Elderly, Risk Factors, Risk Model, Perioperative Care

## Abstract

**Objective:**

This study aimed to estimate the prevalence of frailty among elderly patients undergoing spine surgery at 1 month postoperatively, and develop a perioperative risk model to support targeted medical prevention strategies and facilitate the early identification of high-risk patients.

**Methods:**

This prospective study included 206 elderly patients scheduled for spine surgery between June and August 2023. Frailty was assessed using the FRAIL Scale at admission and one month postoperatively. Data collected included general information, clinical data, laboratory parameters, and pre- and postoperative Athens Insomnia Scale (AIS) scores. Based on the FRAIL Scale scores at one month postoperatively, patients were categorized into a non-frailty group (*n* = 91) and a frailty group (*n* = 115). Univariate analyses were conducted to identify variables associated with postoperative frailty. Least Absolute Shrinkage and Selection Operator (LASSO) regression was employed to identify independent risk predictors A risk model was constructed. Model performance was evaluated using Receiver Operating Characteristic (ROC) analysis, calibration curves, decision curve analysis, discrimination metrics (C-index), and the Hosmer–Lemeshow goodness-of-fit test.

**Results:**

The incidence of frailty and pre-frailty one month after spine surgery was 55.8%. Univariate analysis revealed significant differences between the two groups in age, preoperative frailty score, anesthesia duration, operation duration, total hospitalization costs, preoperative glycated hemoglobin (HbA1c), length of hospital stay, and incidence of postoperative complications (all *P* < 0.05). Multivariate analysis identified age, length of hospital stay, preoperative frailty score, operation duration, anesthesia duration, pre- and postoperative AIS scores, and the number of perioperative medications as independent risk factors (all *P* < 0.05). The LASSO-based risk model demonstrated excellent discrimination, with an area under the curve of 0.958 (95% CI: 0.931–0.985) and a C-index exceeding 0.90. The Hosmer–Lemeshow test indicated good calibration (*P* = 0.109).

**Conclusion:**

The incidence of perioperative frailty is high among elderly patients undergoing spine surgery. A combination of demographic, surgical, and clinical factors contributes to postoperative frailty risk. The perioperative risk model developed in this study demonstrates excellent performance and can serve as a reference for the early identification of high-risk patients and the implementation of targeted preventive strategies.

**Supplementary Information:**

The online version contains supplementary material available at 10.1186/s12891-026-09934-3.

## Introduction

Spine surgery is widely performed for the treatment of degenerative diseases, traumatic fractures, infectious disorders, and structural deformities, and remains a cornerstone of management for these conditions. However, elderly patients present a distinct clinical challenge. Age-related declines in physiological reserve, combined with a high prevalence of multimorbidity and polypharmacy, substantially increase perioperative vulnerability. As a result, older patients undergoing spine surgery face elevated risks of postoperative adverse reactions, including nausea, vomiting, rigors, and dizziness. Moreover, postoperative delirium has been reported in 15% to 53% of this population [1], underscoring the magnitude of perioperative risk.

Frailty is a multidimensional geriatric syndrome caused by cumulative decline across multiple physiological systems and is characterized by reduced resilience to stressors and impaired homeostatic reserve. In elderly patients undergoing spine surgery, frailty is particularly concerning not only during hospitalization but also at 1 month after surgery, when most patients have returned home. At this stage, postoperative frailty or frailty worsening may be difficult for family members to recognize, yet it can substantially impair recovery, reduce functional independence, hinder rehabilitation, and adversely affect quality of life. Because these changes often emerge after discharge and outside direct medical supervision, postoperative frailty may be underestimated despite its important prognostic implications. Importantly, frailty is not an inevitable or irreversible consequence of aging. Early identification of patients at risk and timely intervention may reduce postoperative vulnerability and improve recovery outcomes, making postoperative frailty a clinically important and potentially modifiable condition [2].

Despite its clinical importance, frailty assessment in spine surgery lacks a standardized and systematic risk evaluation framework. Existing approaches are often fragmented and insufficiently tailored to the perioperative setting. Accordingly, this study aims to identify patients who are likely to develop postoperative vulnerability or frailty worsening during the perioperative period, thereby informing timely intervention strategies such as perioperative sleep management, medication review, rehabilitation planning, and closer postoperative surveillance.

## Materials and methods

### Study participants

The sample size was estimated using a rule-of-thumb approach for logistic regression analysis. Based on a preliminary review of the literature, approximately 20 candidate independent variables were anticipated. Following conventional recommendations, a minimum of five observations per variable was required. Assuming a reported prevalence of frailty and pre-frailty among elderly inpatients in China of 63.9% [3], and allowing for an anticipated 20% loss to follow-up, the minimum sample size was calculated as

$$\:\mathrm{n}=20\times\:5/0.639/0.8=195.6\:$$

which was rounded up to 196 participants.

Between June and August 2023, a total of 234 elderly patients scheduled for spine surgery were consecutively enrolled. Inclusion criteria were as follows: Inclusion criteria were as follows: (1) age ≥ 60 years; (2) scheduled to undergo spine surgery (specific procedures are listed in the Appendix); (3) provided written informed consent, were conscious, and able to cooperate with the researchers. Exclusion criteria included: (1) severe mental illness, blindness, hearing impairment, or communication barriers; (2) acute cervical spinal cord injury with paralysis; (3) incomplete preoperative examination data or other essential information; (4) transfer to another hospital or loss to follow-up during the study period. This study was conducted in accordance with the Declaration of Helsinki. Ethical approval was obtained from the Medical Ethics committee of NanFang Hospital of Southern Medical University (approval number: NFEC-2024-539). Informed consent to participate was obtained from all participants prior to enrollment. The study did not use consecutive sampling and to provide a more accurate description of participant enrollment.

### Study instruments and data collection

#### General information questionnaire

Data collected included age, sex, educational level, length of hospital stay, number of preoperative medications, smoking status, alcohol use, and total hospitalization costs.

#### Clinical parameters

Data pertaining to the surgical procedure included surgery type, anesthesia method, duration of anesthesia (hours), duration of surgery (hours), intraoperative blood loss (ml), occurrence of postoperative complications (including delirium, deep vein thrombosis, pulmonary infection, urinary tract infection, intestinal obstruction, and heart failure), and the incidence of surgical site infection during hospitalization.

#### Laboratory parameters

Preoperative laboratory values were collected, including Glycated Hemoglobin (HbA1c), Hemoglobin (HGB), Albumin (HSA), C-Reactive Protein (CRP), White Blood Cell count (WBC), and D-dimer (D-D).

#### Frailty assessment

Frailty status was assessed using the FRAIL Scale at admission and one month postoperatively. In a long-term follow-up study using this scale, Ravindrarajah et al. [4]reported its utility in predicting both disability and mortality. After securing translation permission from Professor Morley [5], we adapted the questionnaire into Chinese. This scale comprises five components: Fatigue, Resistance (ability to climb one flight of stairs), Ambulation (ability to walk one block), Illnesses (number of specific comorbidities), and Loss of Weight. Each component is scored 0 (absent) or 1 (present), yielding a total score ranging from 0 to 5 [6]. A total score of 0 indicates robust health, 1–2 indicates pre-frailty, and ≥ 3 indicates frailty. For this study, patients were dichotomized into a non-frailty group (score = 0) and a frailty group (score = 1–5). The Chinese version of the FRAIL Scale has demonstrated a Cronbach’s α of 0.68–0.75, split-half reliability of 0.73, test-retest reliability of 0.78, and content validity of 0.92.

In addition to the primary analysis based on postoperative frailty status, we performed an additional analysis according to the change in FRAIL score from baseline to 1 month after surgery. Patients were classified into a frailty score increase group and a non-increase group, and the predictive performance of the model for postoperative frailty progression was further evaluated.

#### Sleep assessment

Sleep quality was evaluated using the Athens Insomnia Scale (AIS) one day before surgery and on the second postoperative day. The AIS consists of 8 items: sleep induction, nighttime awakenings, final awakening earlier than desired, total sleep duration, overall sleep quality, daytime well-being, daytime functioning, and daytime sleepiness. Each item is rated from 0 (no problem) to 3 (severe problem), resulting in a total score range of 0–24. According to standard international interpretation, a total score < 4 suggests no sleep disturbance, a score of 0–6 indicates borderline insomnia, and a score > 6 confirms insomnia, with higher scores reflecting poorer sleep quality [7]. The scale reports a Cronbach’s α of 0.82–0.89, split-half reliability of 0.78, test-retest reliability of 0.85, and content validity of 0.94.

#### Delirium risk assessment

Delirium risk was assessed on the second postoperative day using the 4’A’s Test (4AT). This scale was developed by Alasdair MacLullich et al. [8] in 2011 and is designed for delirium assessment in older patients. This rapid assessment tool evaluates four domains: Alertness (normal: 0 points, abnormal: 4 points), Abbreviated Mental Test (no errors: 0 points, 1 error: 1 point, ≥ 2 errors: 2 points), Attention (able to name ≥ 7 months backwards: 0 points, makes errors: 1 point, unable: 2 points), and Acute change or fluctuating course (absent: 0 points, present: 4 points). The total score is interpreted as follows: 0 indicates normal, 1–3 suggests cognitive impairment, and > 4 indicates delirium. The 4AT has a reported Cronbach’s α of 0.72–0.78, split-half reliability of 0.76, test-retest reliability of 0.85, and content validity of 0.91.

### Data collection and quality control

Investigators involved in the study received standardized training. Only those who successfully completed the training were permitted to administer questionnaires using uniform, scripted instructions. After informed consent was obtained, participants completed the questionnaires independently. For participants unable to do so, the investigators provided neutral, standardized descriptions of the questionnaire items and recorded the responses verbatim, avoiding any leading or interpretive guidance. Questionnaires displaying patterned responses, uniform answers across items, or substantial missing data were considered invalid and excluded from analysis.

Post-discharge follow-up was conducted by two dedicated nurses who completed centralized training. Prior to undertaking follow-up responsibilities, they were required to pass monthly simulated follow-up assessments and achieve achieving an inter-rater reliability Kappa coefficient of at least 0.85. Information collected during follow-up was cross-validated against the hospital electronic medical record system and backend application data before final entry into the study database. The assessor performing the 1-month postoperative evaluation was blinded to the patients’ baseline frailty status and other clinical information.

### Least Absolute Shrinkage and Selection Operator (LASSO) regression analysis

The candidate predictors were examined using Employing Least Absolute Shrinkage and Selection Operator (LASSO) cox regression analysis with ten-fold cross-validation via “glmnet” package of R, and a predictive risk coefficient model key predictors with significant non-zero coefficients was developed.

Internal validation was performed using bootstrap resampling with 1,000 repetitions. In each bootstrap sample, the entire LASSO modeling procedure, including 10-fold cross-validation for tuning parameter selection, was repeated. Model optimism was estimated as the mean difference between discrimination in the bootstrap sample and discrimination of the same bootstrap-derived model when applied to the original dataset. The optimism-corrected AUC was calculated by subtracting the estimated optimism from the apparent AUC.

### Statistical analysis

Data analysis was performed using SPSS software (version 22.0). Missing data were addressed using complete-case analysis. After predictor selection, individuals with missing values in variables retained in the final model were excluded from regression modeling. Because the proportion of missingness was limited, no multiple imputation was performed. Continuous variables were tested for normality and are presented as mean ± standard deviation. Comparisons between groups were conducted using independent samples t-tests, while within-group comparisons utilized paired samples t-tests. Categorical data are expressed as percentages (%), and group comparisons were made using the Chi-square (χ²) test. Internal validation of the risk model was performed using the study cohort data. This involved constructing Receiver Operating Characteristic (ROC) curve, plotting calibration curves and decision curves. The model’s diagnostic performance was assessed using likelihood ratio tests, its discriminative ability was evaluated via the Concordance index (C-index), and its goodness-of-fit was tested using the Hosmer-Lemeshow test. A P-value of less than 0.05 was considered statistically significant.

## Results

### Univariate analysis of elderly patients undergoing spine surgery

Twenty-eight cases were excluded due to loss to follow-up at 1 month postoperatively. Among the 206 elderly patients undergoing spine surgery, the combined incidence of frailty and pre-frailty at one month postoperatively was 55.83%. Total 34 candidate predictors were initially included in the analysis. Comparative analyses between the frailty and non-frailty groups identified statistically significant differences (*P* < 0.05) across several variables, including age, preoperative frailty score, duration of anesthesia, duration of surgery, total hospitalization costs, preoperative glycated hemoglobin (HbA1c), length of hospital stay, and the incidence of postoperative complications. Details are presented in Table 1.

**Table 1:** Comparison between frailty and non-frailty groups

Variables	Non-frailty group (*n* = 91)	Frailty group(*n* = 115)	*P* value
Age	67.55 ± 5.23	69.69 ± 5.70	0.006*
Gender, n (%)			0.821
Male	41(19.9%)	50(24.3%)	
Female	50(24.3%)	65(31.6%)	
Smoking, n (%)			0.828
No	79(38.3%)	101(49%)	
Yes	12(5.8%)	14(6.8%)	
Alcohol drinking, n (%)			0.689
No	85(41.3%)	110(53.4%)	
Yes	6(2.9%)	5(2.4%)	
Preoperative frailty score	0.33 ± 0.56	2.21 ± 1.13	< 0.001*
Preoperative hypertension, n (%)			
No	75(36.4%)	92(44.7%)	
Yes	16(7.8%)	23(11.2%)	
SPO2 ≤ 90% occurred during hospitalization, n (%)			
No	90(43.7%)	108(52.4%)	
Yes	1(0.5%)	7(3.4%)	
Surgical method, n (%)			0.499
Endoscopic lumbar discectomy	2(1%)	4(1.9%)	
Vertebroplasty	11(5.3%)	9(4.4%)	
Spinal nerve root block	2(1%)	3(1.5%)	
Cervical posterior unilateral open-door decompression	12(5.8%)	23(11.2%)	
Anterior cervical interbody fusion	3(1.5%)	1(0.5%)	
Thoracic vertebral lamina resection and decompression	0(0%)	2(1%)	
Lumbar interbody fusion	55(26.7%)	63(30.6%)	
Percutaneous vertebral biopsy	6(2.9%)	10(4.9%)	
Operation duration, min	130.74 ± 50.81	157.01 ± 73.96	0.003*
Anesthesia method, n (%)			0.571
General anesthesia	69(33.5%)	91(44.2%)	
Local anesthesia	22(10.7%)	24(11.7%)	
Anesthesia duration (min)	276.57 ± 92.62	315.44 ± 106.74	0.015*
Intraoperative blood loss (ml)	163.13 ± 135.39	202.67 ± 162.98	0.109
Incision infection during hospitalization, n (%)			1.000
No	89(43.2%)	113(54.9%)	
Yes	2(1%)	2(1%)	
Postoperative complications, n (%)			0.012*
No	89(43.2%)	102(49.5%)	
Yes	2(1%)	13(6.3%)	
Total hospitalization expenses (yuan)	41,570 ± 19,190	49,050 ± 26,420	0.024*
Length of hospital stay, (day)	8.47 ± 3.25	11.99 ± 6.10	< 0.001*
Preoperative fasting blood glucose (mmol/L)	5.31 ± 2.03	5.77 ± 2.38	0.138
Preoperative glycated hemoglobin (%)	5.92 ± 0.98	6.64 ± 1.56	< 0.001*
Preoperative glomerular filtration rate (ml/min)	81.51 ± 15.87	78.50 ± 20.43	0.237
Preoperative transaminase ratio	1.24 ± 0.74	2.30 ± 9.21	0.225
Preoperative creatinine (umol/L)	70.26 ± 20.03	77.46 ± 49.10	0.156
Preoperative hemoglobin (g/L)	128.78 ± 18.12	123.46 ± 18.67	0.041*
Preoperative albumin (g/L)	39.23 ± 3.30	37.52 ± 4.70	0.003*
Preoperative C-reactive protein (mg/L)	4.34 ± 8.00	11.35 ± 22.53	0.002*
Preoperative procalcitonin (10ng/ml)	0.03 ± 0.03	0.07 ± 0.15	0.008*
Total preoperative white blood cell count (10^9/L)	6.89 ± 3.00	6.63 ± 2.40	0.497
Preoperative D-dimer (ug/mL FEU)	3.07 ± 17.46	1.78 ± 3.48	0.450

Data are presented as mean ± standard deviation for continuous variables and number (percentage) for categorical variables. * denotes a statistically significant difference between groups with *P* < 0.05

### ROC and LASSO analysis results of the 206 patients

**Fig. 1:**
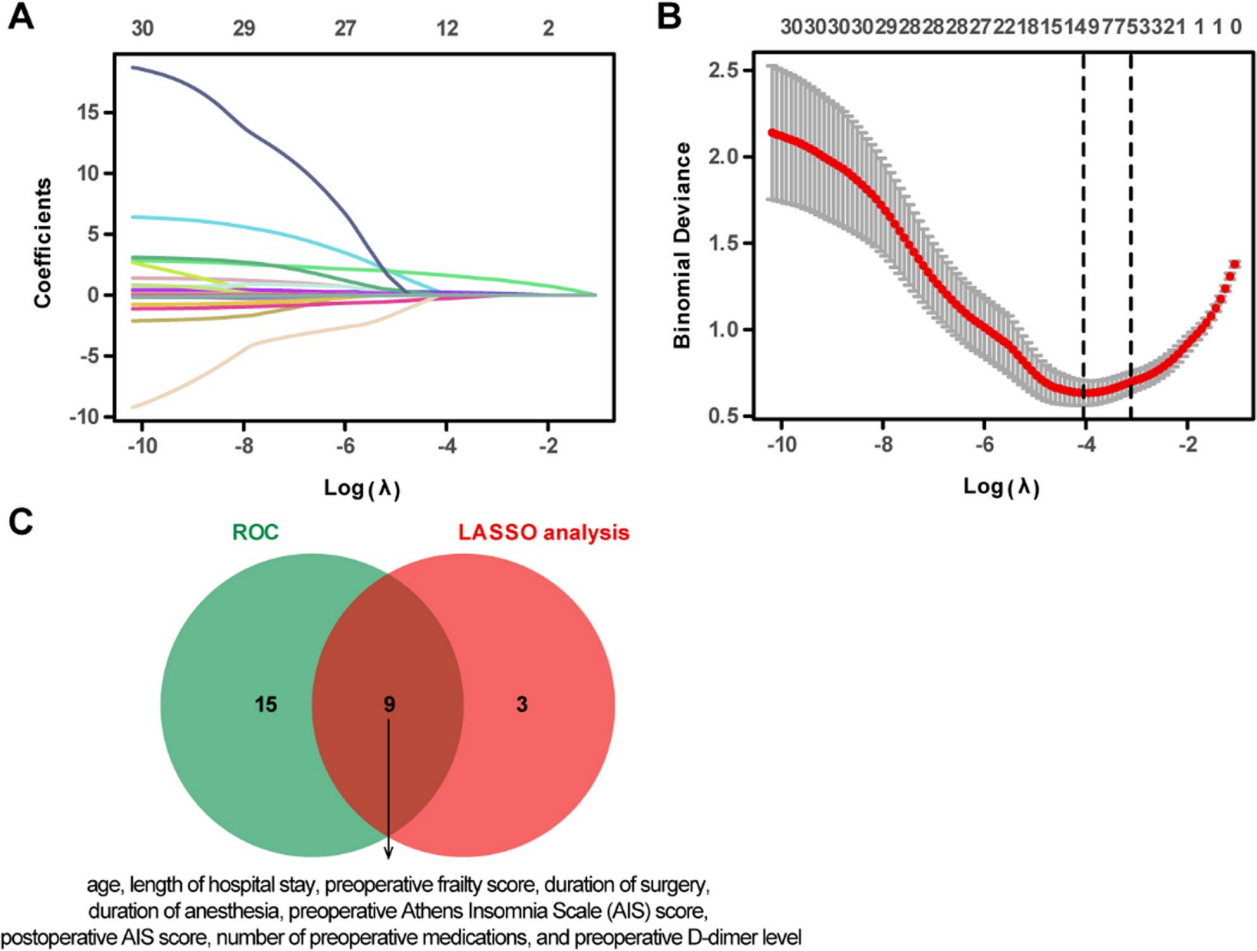
Identification of predictive features. **A** Trajectory of LASSO coefficients. **B** Feature selection in LASSO model. **C** Overlap of significant variables from ROC (AUC > 0.5) and LASSO (non-zero coefficients) analyses. LASSO, Least Absolute Shrinkage and Selection Operator. ROC, Receiver Operating Characteristic. AUC, Area Under the Curve

Results from the ROC and LASSO regression analyses demonstrated that age, length of hospital stay, preoperative frailty score, duration of surgery, duration of anesthesia, preoperative Athens Insomnia Scale (AIS) score, postoperative AIS score, number of preoperative medications, and preoperative D-dimer level were identified as independent risk factors for perioperative frailty in the elderly patients undergoing spine surgery (*P* < 0.05). 12 variables for LASSO regression analyses with non-zero coefficients at λ(min) were retained for subsequent analysis. Details are presented in Table 2; Fig. 1.

**Table 2:** Independent Risk Factors Identified by LASSO and ROC Analyses (*n* = 206)

Variables	ROC Analysis			LASSO Analysis
	Predicted Outcome	Area Under the Curve	Confidence Interval	λ(min)
Age	Positive	0.614	0.537–0.691	0.068
Gender	Positive	0.508	0.439–0.577	0
Smoking	Positive	0.495	0.449–0.541	0
Alcohol consumption	Positive	0.489	0.457–0.521	0.13
Preoperative frailty score	Positive	0.906	0.866–0.945	1.64
Surgical approach	Positive	0.496	0.424–0.567	-0.29
Operation duration	Positive	0.591	0.513–0.668	8.95E-05
Anesthesia method	Positive	0.517	0.459–0.574	0
Anesthesia duration	Positive	0.642	0.556–0.729	0.00021
Intraoperative blood loss	Positive	0.58	0.490–0.670	0
Surgical site infection during hospitalization	Positive	0.498	0.478–0.517	0
Postoperative complications	Positive	0.546	0.513–0.578	0
Total hospitalization cost	Positive	0.596	0.519–0.674	0
Length of hospital stay	Positive	0.684	0.612–0.756	0.24
Postoperative delirium	Positive	0.498	0.478–0.517	0
Preoperative Athens Insomnia Scale	Positive	0.597	0.517–0.676	0.024
Postoperative Athens Insomnia Scale	Positive	0.574	0.495–0.653	0.10
Preoperative medication types	Positive	0.763	0.715–0.811	0.01
Preoperative glycated hemoglobin (%)	Positive	0.667	0.593–0.741	0
Preoperative hemoglobin (g/L)	Negative	0.584	0.506–0.662	0
Preoperative albumin (g/L)	Negative	0.639	0.563–0.714	0
Preoperative C-reactive protein (mg/L)	Positive	0.588	0.510–0.666	0
Preoperative total white blood cell count (10^9/L)	Positive	0.494	0.414–0.574	0
Preoperative D-dimer (µg/mL)	Positive	0.546	0.465–0.627	-0.004

### Development of a LASSO-based risk model for perioperative frailty in elderly spine surgery patients

**Fig. 2:**
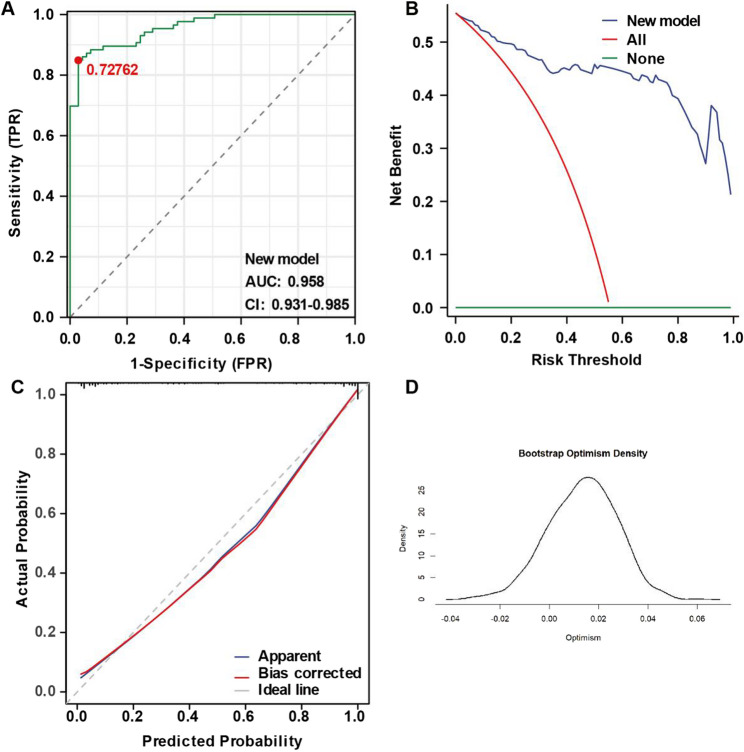
Model performance validation. **A** ROC curve. **B** Decision curve analysis. **C** Calibration plot. ROC, Receiver Operating Characteristic. **D** The distribution of optimism estimated from 1,000 bootstrap resamples

In light of the preceding analyses, preoperative D-dimer was excluded from the subsequent model because it exhibited inconsistent associations with the outcome across the ROC (positive, AUC = 0.546) and LASSO regression (λ(min) = -0.004) analyses. A revised risk model was therefore constructed using LASSO regression to predict the risk of frailty at one month postoperatively in elderly patients undergoing spine surgery. The risk coefficients for the predictors in this final model are reported in Table 3, and the curves evaluating its predictive performance are presented in Fig. 2.

**Table 3:** Coefficients of frailty risk predicted by combined ROC analysis one month after surgery

Multivariate Model Variables	Coefficient
Intercept	-11.731526
Age	0.09107803
Length of hospital stay	0.23575707
Preoperative frailty score	1.76981079
Operation duration	0.00115912
Anesthesia duration	0.00061745
Preoperative Athens Insomnia Scale	0.05273007
Postoperative Athens Insomnia Scale	0.17075617
Preoperative medication types (> 5 types)	0.20871974

### Validation of the LASSO model diagnostic performance

Internal validation was performed using the study cohort. The ROC analysis demonstrated that the LASSO-based risk model achieved an Area Under the Curve (AUC) of 0.958 (95% CI: 0.931–0.985), indicating excellent predictive ability. The likelihood ratio test was statistically significant (*P* < 0.05), confirming that the model provided explanatory power beyond the null. Consistent with this finding, the Concordance Index (C-index) exceeded 0.9, reflecting high predictive accuracy. Model calibration was satisfactory, as the Hosmer-Lemeshow goodness-of-fit test yielded a non-significant result (*P* > 0.05), indicating close agreement between predicted and observed outcomes. Detailed results are presented in Tables 4, 5 and 6; Fig. 2A-C.

**Table 4:** Diagnostic results of decision curve analysis (DCA)

Variable	Type	Number	Coefficient β	OR	95% Confidence Interval	*P*-value
New model	Numerical variable	155	1.24	3.45	2.32–5.13	< 0.001

**Table 5:** Calibration curve analysis of predicted versus observed probabilities

Variable	Coefficient β	OR	95% Confidence Interval	*P*-value
New model	1.24	3.45	2.32–5.14	< 0.001

**Table 6:** Goodness-of-fit evaluation of the risk model via calibration curves

Evaluation Aspect	Evaluation Content	Statistic	*P*-value
Model test	Likelihood ratio test	χ²=133.6	0
Discrimination evaluation	C-index	0.96 (0.93–0.99)	< 0.001
Calibration evaluation	Goodness-of-fit test	χ²=13.08	0.11

Besides, the apparent C-index of the model was 0.958. After 1,000 bootstrap resamples, the estimated optimism was 0.0135, yielding an optimism-corrected AUC of 0.9446. Figure 2D shows the distribution of optimism estimated from 1,000 bootstrap resamples. The mean optimism was small, indicating limited overfitting and stable model performance.

In a sensitivity analysis using the standard three-category FRAIL classification (robust / pre-frail / frail), ordinal logistic regression demonstrated that the composite risk score was strongly associated with progression to a worse frailty category (β = 0.895, OR = 2.45, *P* < 0.001). Each one-unit increase in the risk score was associated with a 2.45-fold increase in the odds of belonging to a higher frailty category (Table 7).

**Table 7:** Sensitivity Analysis Using the Standard Three-Category FRAIL Classification (Robust / Pre-frail / Frail)

Variable	Value	Std.Error	OR	95% Confidence Interval	t.value	*p*
New model	0.90	0.11	2.45	2.01–3.09	8.18	< 0.001

**Fig. 3:**
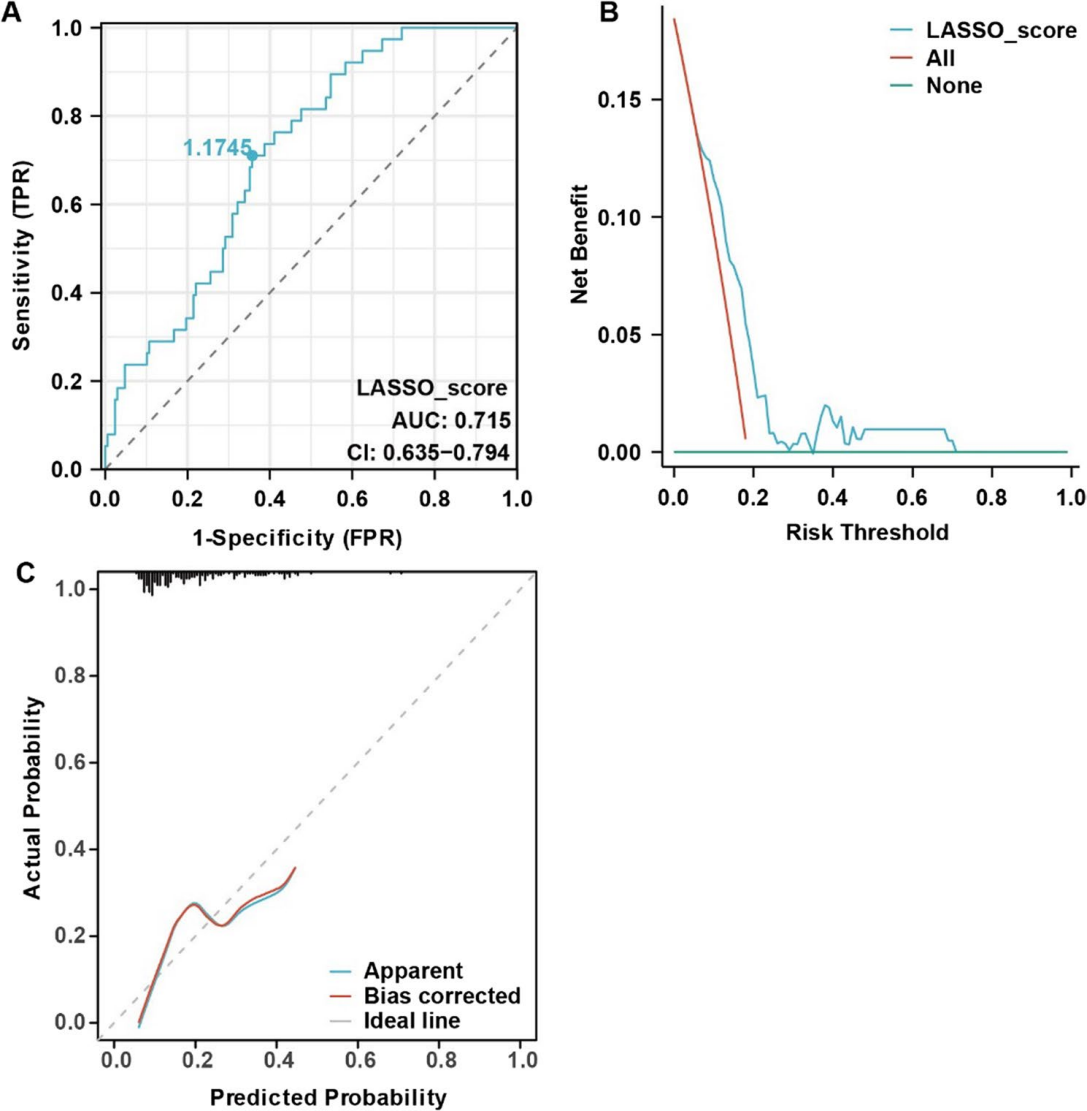
Model performance validation stratified by increased FRAIL score. **A** ROC curve. **B **Decision curve analysis. **C** Calibration plot. ROC, Receiver Operating Characteristic

To further assess whether the model was associated with perioperative frailty progression rather than merely reflecting baseline frailty, patients were additionally categorized according to whether their FRAIL score increased at 1 month after surgery. The model also demonstrated satisfactory predictive performance for identifying patients with postoperative frailty score increase (AUC, 0.715, Fig. 3A), suggesting its potential utility in recognizing elderly patients at risk of postoperative deterioration. The Concordance Index (C-index) exceeded 0.715, reflecting high predictive accuracy. Model calibration was satisfactory, as the Hosmer-Lemeshow goodness-of-fit test yielded a non-significant result (*P* > 0.05), indicating close agreement between predicted and observed outcomes. Detailed results are presented in Tables 8 and 9; Fig. 3A-C.

**Table 8:** Calibration curve analysis of predicted versus observed probabilities stratified by increased FRAIL score

Variable	Coefficient β	OR	95% Confidence Interval	*P*-value
New model	0.22	1.25	1.12–1.39	< 0.001

**Table 9:** Goodness-of-fit evaluation of the risk model via calibration curves stratified by increased FRAIL score

Evaluation Aspect	Evaluation Content	Statistic	*P*-value
Model test	Likelihood ratio test	χ²= 17.75	< 0.001
Discrimination evaluation	C-index	0.72 (0.63–0.80)	< 0.001
Calibration evaluation	Goodness-of-fit test	χ²= 11.59	0.17

## Discussion

Frailty is fundamentally characterized by diminished physiological reserve and impaired stress response capacity, with its pathogenesis involving multisystem homeostatic dysregulation. In elderly patients undergoing spine surgery, the physiological burden imposed by surgical trauma challenges already limited compensatory mechanisms, accelerating endocrine, metabolic, and immunoregulatory dysfunction. This process substantially increases the risk of postoperative disability and other adverse outcomes. Developing a perioperative frailty risk model enables early identification of high-risk populations, provides a scientific basis for targeted interventions, and holds substantial clinical value for mitigating frailty progression and improving postoperative prognosis [9–12].

### High postoperative frailty prevalence and elevated complication rates in the frail group

This study found that the combined incidence of frailty and pre-frailty one month after spine surgery reached 55.8% among elderly patients, indicating a substantial perioperative frailty burden. Compared with non-frail patients, those classified as frail experienced markedly higher rates of postoperative complications, including delirium, deep vein thrombosis, pulmonary infection, urinary tract infection, intestinal obstruction, heart failure, and surgical site infection. These findings are consistent with the report by McCandless et al. [13].

Population aging is accompanied by progressive declines in physiological function and compensatory capacity, particularly in vital organ systems such as the cardiovascular and respiratory systems, which further amplifies vulnerability to perioperative frailty [14]. Prior studies have demonstrated a close association between preoperative frailty and adverse postoperative outcomes [15]. Consistent with this evidence, our results showed that frail patients had significantly longer hospital stays and higher rates of postoperative complications, aligning with the conclusions of Niu Ogata et al. [16]. and Maed et al. [17].Mechanistically, frailty is often accompanied by immune dysfunction and reduced mobility, frequently necessitating prolonged bed rest, which increases susceptibility to complications such as pulmonary infection and, in turn, prolongs hospitalization [18].

### Independent risk factors for perioperative frailty

Prior studies have identified multiple determinants of perioperative frailty in elderly surgical patients. McIsaac et al. [19] and Lin et al. [20] identified multiple comorbidities, polypharmacy, lack of regular exercise, and elevated C-reactive protein are significant contributors. Dent et al. [21] characterized frailty as a complex clinical syndrome closely associated with advanced age. Hewitt et al. [22] further demonstrated that prolonged surgical duration increases the risk of perioperative frailty, while Fanelli et al. [23] and Sonawane et al. [24]noted that regional anesthesia has smaller impact on physiological function. Previous evidence suggests that age, polypharmacy, inflammatory response, and anesthesia technique are closely linked to frailty progression. Collectively, existing evidence indicates that age, medication burden, inflammatory response, and anesthetic factors are central to frailty progression.

Building on these literatures, our findings suggest that perioperative frailty risk is driven by the combined effects of baseline frailty status, sleep disturbances as measured by the Athens Insomnia Scale, surgical stress, metabolic dysregulation (including abnormal HbA1c and D-dimer levels), and prolonged hospitalization. These factors appear to act synergistically, substantially increasing the likelihood of postoperative frailty in elderly patients undergoing spine surgery.

Univariate analyses identified significant between-group differences (*P* < 0.05) in age, preoperative frailty score, anesthesia duration, operation duration, total hospitalization costs, preoperative glycated hemoglobin, length of hospital stay, and postoperative complication rate. Subsequent multivariate analysis further demonstrated that age, length of hospital stay, preoperative frailty score, operation duration, anesthesia duration, pre- and postoperative Athens Insomnia Scale (AIS) scores, and the number of preoperative medications were independent risk factors for perioperative frailty.

Notably, preoperative D-dimer exhibited inconsistent associations with postoperative frailty across analytical approaches. Although ROC analysis suggested acceptable discriminatory performance in the training set, cross-validation within the LASSO framework indicated a negative contribution to predictive accuracy in the validation process. Inconsistency is likely attributable to strong correlations between D-dimer and inflammatory markers such as C-reactive protein and white blood cell count, reflecting its dual involvement in systemic inflammation and neurological injury [25]. leading to high multicollinearity with variables like WBC. Correlation analysis showed that D-dimer was positively correlated with WBC (supplementary Fig. 1, *R* = 0.563, *p* < 0.05), indicating partial but not uniform overlap with inflammation-related markers. Accordingly, D-dimer was excluded, and clinically accessible predictors with lower collinearity were retained to improve model stability, precision, and generalizability.

### Reliability and scientific validity of the frailty risk risk model

The frailty risk model developed using LASSO regression demonstrated excellent discriminative performance, with an area under the ROC curve of 0.958 (95% CI: 0.931–0.985). Model calibration was satisfactory, as indicated by a non-significant Hosmer-Lemeshow goodness-of-fit test (*P* > 0.05). By integrating multidimensional risk indicators, the model supports preoperative risk stratification, and facilitates informed clinical decision-making across the preoperative, intraoperative, and postoperative phases. Specifically, it enables early identification of high-risk patients, optimization of anesthesia management, and timely implementation of individualized preventive strategies. Collectively, these features underscore the model’s potential clinical utility in improving perioperative outcomes among elderly patients undergoing spine surgery. Importantly, the association remained robust when the FRAIL Scale was analyzed using its conventional three-category structure rather than dichotomization. This finding supports the stability and clinical relevance of the proposed risk score.

To address the concern that postoperative frailty status may be largely driven by baseline frailty, we additionally evaluated the model against postoperative increase in FRAIL score. This supplementary analysis suggests that the model may capture not only baseline vulnerability but also the risk of postoperative frailty worsening, which is of greater clinical relevance for perioperative intervention planning.

## Limitations

As this was a single-center study, external validation using larger, multicenter cohorts is required to confirm the model’s robustness and generalizability. Another limitation of this study is that the final model included postoperative variables, including postoperative AIS score and length of hospital stay. Therefore, the model is not suitable as a purely preoperative risk stratification tool, and future studies should develop and validate a model based solely on preoperative predictors for broader clinical applicability. The current study used multivariable regression, LASSO regression, and ROC analysis to evaluate the model, and that a nomogram was not additionally included in this version. Finally, the FRAIL scale, which, although practical for repeated perioperative assessment, may be less comprehensive than frailty index-based instruments and may limit direct comparison with other spine surgery studies. In addition, the dichotomization of FRAIL scores should be interpreted cautiously, and future studies should compare different frailty tools in larger independent cohorts.

## Conclusion

Frailty vulnerability at 1 month after spine surgery is clinically important in elderly patients, particularly after discharge when it may be difficult for family members to recognize but may still have substantial effects on recovery and functional independence. The present study identified several associated factors and developed a preliminary perioperative risk model that may help support earlier recognition and intervention. Further validation is required before broader clinical application.

## Supplementary Information

Supplementary Material 1.

## Data Availability

The datasets used and/or analyzed during the current study are available from the corresponding author upon reasonable request.
